# Drugs and Targets in Fibrosis

**DOI:** 10.3389/fphar.2017.00855

**Published:** 2017-11-23

**Authors:** Xiaoyi Li, Lixin Zhu, Beibei Wang, Meifei Yuan, Ruixin Zhu

**Affiliations:** ^1^Department of Gastroenterology, School of Life Sciences and Technology, Shanghai East Hospital, Tongji University, Shanghai, China; ^2^Department of Pediatrics, Digestive Diseases and Nutrition Center, State University of New York at Buffalo, Buffalo, NY, United States; ^3^Genome, Environment and Microbiome Community of Excellence, State University of New York at Buffalo, Buffalo, NY, United States; ^4^Center for Drug Discovery, SINO High Goal Chemical Technology Co., Ltd., Shanghai, China

**Keywords:** fibrosis, drug, target, pathological mechanism, pharmacology

## Abstract

Fibrosis contributes to the development of many diseases and many target molecules are involved in fibrosis. Currently, the majority of fibrosis treatment strategies are limited to specific diseases or organs. However, accumulating evidence demonstrates great similarities among fibroproliferative diseases, and more and more drugs are proved to be effective anti-fibrotic therapies across different diseases and organs. Here we comprehensively review the current knowledge on the pathological mechanisms of fibrosis, and divide factors mediating fibrosis progression into extracellular and intracellular groups. Furthermore, we systematically summarize both single and multiple component drugs that target fibrosis. Future directions of fibrosis drug discovery are also proposed.

## Introduction

Fibrosis, characterized by excess accumulation of extracellular matrix (ECM), is a common pathological process in many chronic diseases or injuries. Many irritations trigger the pro-fibrotic responses, including persistent infections, radiation, chemical agents, genetic disorders, and autoimmune diseases. The development of fibrosis is accompanied by the loss of a fraction of resident cells and their replacement by ECM, which would finally lead to tissue remodeling and organ failure. Fibrosis contributes to high morbidity and mortality in many diseases such as dilated cardiomyopathy and idiopathic pulmonary fibrosis (IPF) (Gulati et al., 2013; Hutchinson et al., 2015), and inevitably causes a prominent global clinical burden (Raimundo et al., 2016). For example, a study of medicare population aged 65 years and older showed that the incidence of IPF was around 93.7 cases per 100,000 person-years while the cumulative prevalence increased steadily to 494.5 cases per 100,000 person-years across 2001 to 2011 in US (Raghu et al., 2014). Besides, the mortality of non-alcoholic fatty liver disease (NAFLD) patients with a high probability of fibrosis was 69% higher than those without fibrosis (Kim et al., 2013).

**Figure 1:**
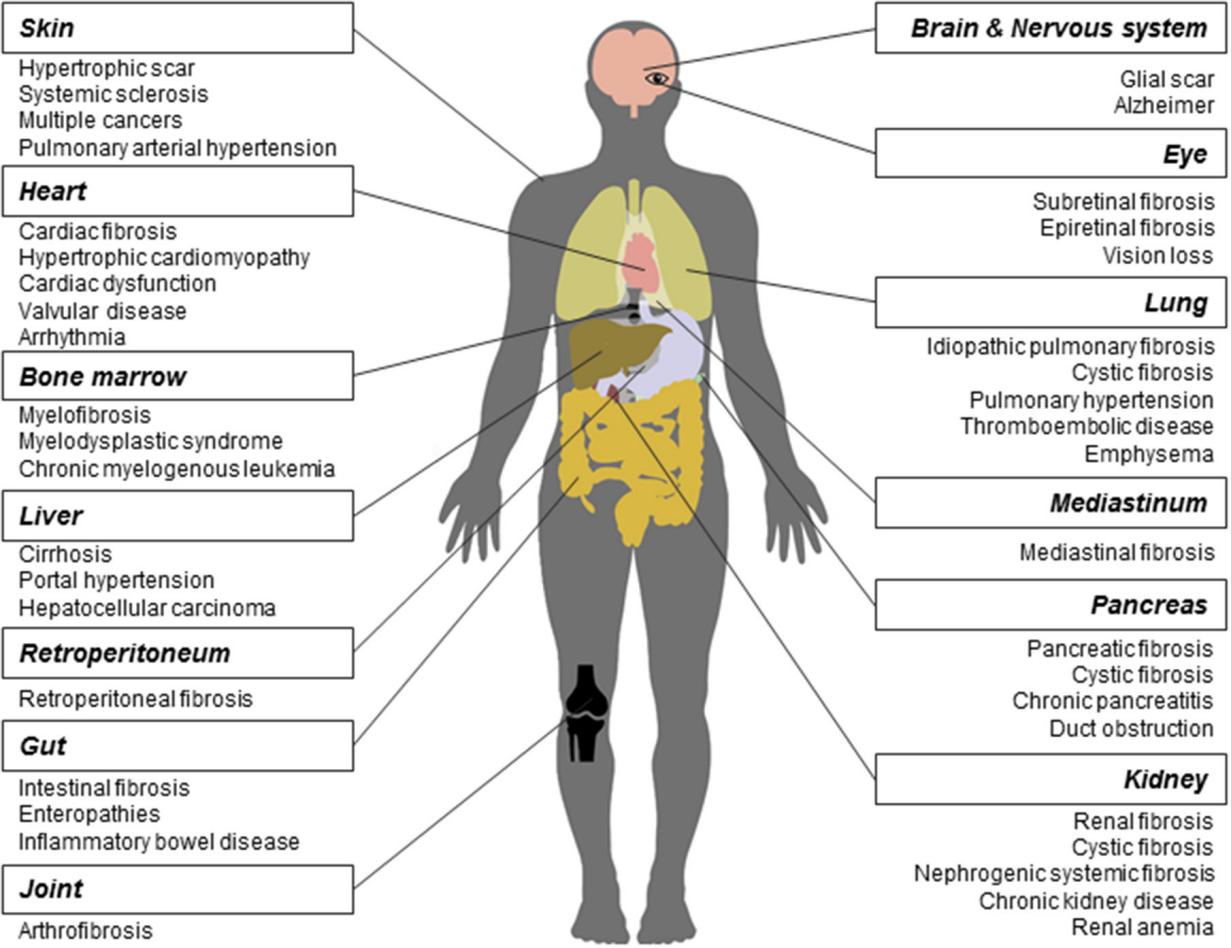
Fibrosis and related diseases in various tissues and organs. Fibrosis is a pathological process that could occur in many tissues and organs and is associated with multiple diseases. Commonly reported fibrosis and associated diseases are listed.

As a long-lasting pathological phenomenon, fibrosis occurs in various tissues and organs (Figure 1), more often in heart, lung, kidney, liver, skin (Rockey et al., 2015), and less frequently in other tissues and organs such as pancreas, intestine, eye (Wynn, 2008), nerve system (Kawano et al., 2012), mediastinum (Parish and Rosenow, 2002), retroperitoneum (Caiafa et al., 2013), joint and tendon (arthrofibrosis).

Fibrosis contributes to the development of many diseases. First, many studies have demonstrated that the core mechanisms in fibrosis across various tissues and organs are similar. Wang and colleagues found that the interaction between transforming growth factor-β(TGF-β) and connective tissue growth factor signaling is required in kidney, liver, and lung fibrosis (Wang Q. et al., 2011). Makarev and colleagues identified a number of common pathways between lung and liver fibrogenesis, such as TGF-β, interleukin-6(IL-6), and integrin-linked kinase signaling (Makarev et al., 2016). Moreover, Wenzke and colleagues detected 90 genes, as well as several networks associated with connective tissue disorders, that play important roles in multi-organ fibrosis including lung, heart, liver, and kidney (Wenzke et al., 2012). On the basis of common pathogenesis across fibroproliferative diseases, some new drugs were proved effective in the treatment of fibrosis across different tissues and organs. For example, Pirfenidone has entered into the phase II clinical trial for treating the systemic sclerosis(SSc) (Khanna et al., 2016) and the phase III for IPF (King et al., 2014), respectively. Interferon drug Actimmune has been evaluated in patients with IPF (Skaria et al., 2015), liver (Muir et al., 2006), and cystic fibrosis (Moss et al., 2005).

Second, in different tissues and organs, multiple fibrotic diseases are related to each other. They are usually triggered by the same irritation and occur simultaneously. For example, heart and kidney together develop fibrosis (cardiorenal fibrosis) owing to the imbalance of natriuretic peptide system pathway and renin angiotensin aldosterone system/TGF-β1 pathway in aging (Sangaralingham et al., 2016). Chronic or acute renal failure may induce nephrogenic systemic fibrosis developed from thickening skin (Reiter et al., 2012) to impaired internal organs. In addition, cystic fibrosis, caused by gene mutation, could widely affect multiple organs, such as lung, kidney, and pancreas. Moreover, fibrosis is frequently a common pathological process in NAFLD and inflammatory bowel disease. Replacement of heart tissues by fibrotic protein could alter the ventricle size and shape, leading to hypertrophic cardiomyopathy (Khan and Sheppard, 2006). Cancers such as hepatocellular carcinoma share a series of risk factors with liver fibrosis (De Minicis et al., 2012).

Here we review the current knowledge on the pathological mechanisms of fibrosis and systematically summarize drugs targeting fibrosis in different fibroproliferative diseases. Future directions for fibrosis drug discovery are also proposed.

## Pathogenesis of fibrosis

**Figure 2:**
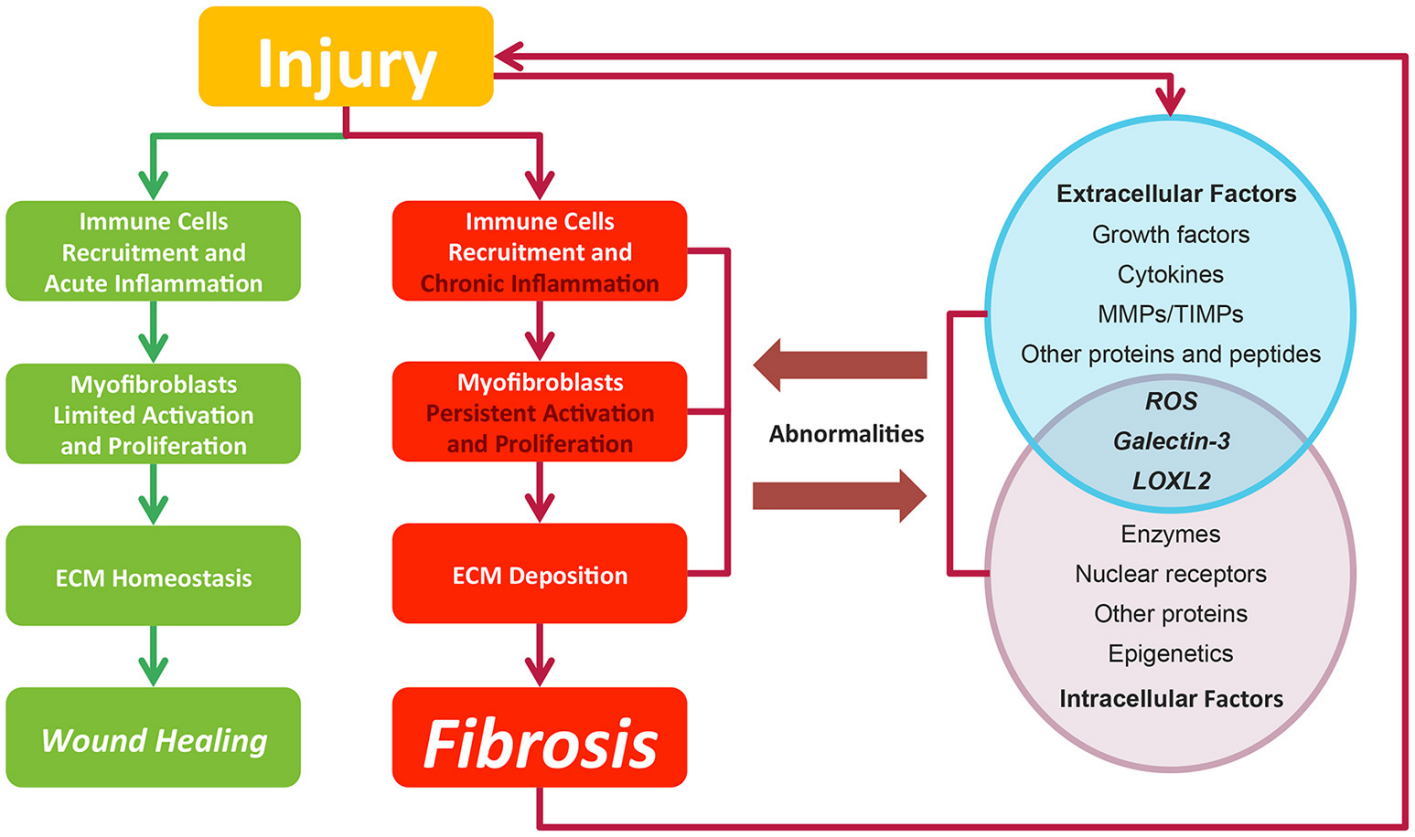
In normal wound healing condition, there is a series of ordered process: injury, immune cell recruitment and acute inflammation, myfibroblasts limited activation, and proliferation and ECM homeostasis, leading to wound closure after injury. While in pro-fibrotic condition, pathological process including chronic inflammation, myfibroblasts persistent activation, and proliferation and ECM deposition leading to fibrosis. Fibrosis itself could result in a secondary assault. Extracellular and intracellular factors interact with each other. Their abnormalities contribute to the fibrosis progression and in return are affected by pathological changes. Green lines and blocks represent normal wound healing while red lines and blocks represent fibrosis progression.

Fibrosis is considered as pathological outcomes of normal wound healing (Figure 2). When injuries occur and epithelial and/or endothelial cells are damaged, pro-inflammatory cytokines are released by the coagulation cascade for immune cell recruitment, mainly neutrophils and macrophages. These recruited immune cells function as the scavenger to remove tissue debris and dead cells, resulting in acute inflammation. Meanwhile, immune cells themselves release factors like chemokines and cytokines to amplify inflammatory reactions. Next, the released factors, such as TGF-β (Thannickal et al., 2003), platelet derived growth factor (PDGF) (Tang et al., 1996), interleukin-13 and interleukin-4 (Hashimoto et al., 2001), induce the limited activation and proliferation of myofibroblasts. Besides resident fibroblasts, myofibroblasts are derived from multiple cells (Hinz et al., 2007), including fibrocytes, epithelial cells via epithelial-mesenchymal transition (EMT), endothelial cells via endothelial-mesenchymal transition, pericytes, and smooth muscle cells related to blood vessels. In liver and pancreas, precursor cells like hepatic stellate cells (HSC) (Moreira, 2007) and pancreatic stellate cells (Apte et al., 2012) could also acquire myofibroblastic phenotype. Activated myofibroblasts migrate to injury sites, and their abilities to generate cell traction force enable them to stimulate wound closure (Li and Wang, 2011). Then, the balance of ECM synthesis and degradation could be achieved by myofibroblasts, resulting in ECM homeostasis. Finally, immune cells undergo apoptosis and epithelial/endothelial cells proliferate to regenerate injury sites, leading to wound healing.

In contrast to normal wound healing process, the abnormalities of multiple factors could cause fibrosis (Biernacka et al., 2011) (Figure 2). Under some persistent stimuli, the overexpression of factors like pro-inflammatory cytokines or growth factors would overactivate (Kim et al., 2008) and interact with multiple kinases or nuclear receptors. The deficiencies of some factors would also contribute to fibrosis progression (Allen and Spiteri, 2002). Then, the abnormal signaling sustains to switch normal wound healing process to pro-fibrotic process, acting on the recruitment of excess immune cells, the induction of the myofibroblasts activation and proliferation and the promotion of ECM production. Pro-fibrotic process also promotes the activation of these factors conversely, thereby amplifying inflammatory responses and causing chronic inflammation. Finally, the sustained myofibroblasts activation would generate masses of ECM and tilt the balance in favor of synthesizing ECM to produce fibrosis. Furthermore, the pro-fibrotic process itself could contribute to secondary injury to the wound and cause a chronic vicious circle of pathological responses.

Below we divide factors modulating fibrosis progression into extracellular and intracellular groups, and discuss how they influence fibrosis progression.

### Extracellular factors mediating the progression of fibrosis

The majority of the fibrosis-related extracellular factors are receptor-binding ligands, such as growth factors and cytokines. These factors target adjacent and distant cells in autocrine, paracrine, or endocrine signaling pathways. Then they bind to specific receptors on cell membrane and trigger the intracellular signaling, leading to pro-fibrotic cellular responses. Other extracellular factors, mainly enzymes such as matrix metalloproteinases (MMPs) could degrade ECM to prevent its excessive accumulation.

Growth factors contain a huge family of proteins that stimulate cell growth and proliferation. They are secreted by fibroblasts, immune cells, and epithelial/endothelial cells, and are able to orchestrate cellular responses. While epithelium/endothelium are damaged, cells in these tissues massively upregulate the production of growth factors to promote the proliferation of immune cells and fibroblasts. Among growth factors, TGF-β is the “master” modulator in fibrogensis (Meng et al., 2016), as it could provoke fibrosis through SMAD-dependent pathway (Lan, 2011) and SMAD-independent pathway related to a number of other pro-fibrotic reactions (Zhang, 2009). TGF-β signaling cascade results in differentiation of effector cells via inducing the expression of myofibroblasts hallmark α-hallmar muscle actin(α-SMA) (Sebe et al., 2008). In addition, TGF-β signaling leads to the transcription of collagen I and III genes (Fine and Goldstein, 1987; Chen et al., 1999) contributing to ECM accumulation. Interacting with TGF-β, many other growth factors have distinct roles in pro-fibrotic process. For example, PDGF induces HSC proliferation and type I collagen expression via downstream focal adhesion kinase/phosphoinositide 3-kinase/protein kinase B signaling (Reif et al., 2003). On the contrary, some growth factors have anti-fibrotic property, such as hepatocyte growth factor (HGF), the overexpression of which alleviates fibrosis in cardiomyopathic hamster through activation of MMP-1 and urokinase-type plasminogen activator (Taniyama et al., 2002). Excessive cytokines are usually secreted by immune cells, such as macrophages, neutrophils, and T cells in inflammation phase of wound healing. Compared with growth factors, cytokines tend to act as cell signaling transmitters to augment immunological responses and then lead to inflammation. One major type of cytokines is chemokines, which guide the recruitment of immune cells and fibroblasts to injury sites. Different immune cells are recruited by different chemokines. Neutrophils could be recruited by chemokine (C-X-C motif) ligand 1 and chemokine (C-X-C motif) ligand 8 by binding glycosaminoglycans with receptors in slightly different ways (Sawant et al., 2016). Chemokine (C-C motif) ligand 5 has been a major factor to induce the migration of HSC in liver fibrosis (Seki et al., 2009). Another type of cytokines is T cell cytokines, which are mainly secreted by activated T lymphocytes. They include interleukins, interferons and tumor necrosis factors (TNF), mediating adaptive immune responses and inflammation that might promote fibrosis. For example, IL-6 has been reported to shift the tissue repair to a chronic inflammatory state by signal transducer and activator of transcription 3(STAT3) signaling pathway in peritoneal fibrosis (Fielding et al., 2014). In contrast, some cytokines have anti-fibrotic effects. It has been reported that interferon-γ down-regulated the Adenosine A2A receptor signaling to prevent the production of type I collagen in HSC (Block and Cronstein, 2010). Moreover, some T cell cytokines, such as TNF-α, exhibit two-sided effects, pro-fibrotic or anti-fibrotic effect depending on the alternative status of macrophages and micro-environment (Redente et al., 2014).

MMPs are the extracellular endopeptidases degrading ECM including collagens, proteoglycans, laminins, and fibronectin. Tissue inhibitor of metalloproteinases (TIMPs) work as the inhibitors of MMPs. The balance of MMPs and TIMPs modulates the process throughout fibrosis development, including the formation of multiple cell injuries, the activation of latent cytokines and myofibroblasts and mainly, the maintenance of the homeostasis of ECM (Giannandrea and Parks, 2014). Some MMPs have pro-fibrotic functions whereas some have anti-fibrotic according to cell types and phases. The dysregulation of MMP-19 has been proved to cause the degradation of normal liver ECM and initiate liver injury (Jirouskova et al., 2012). Conversely, MMP-2 has been reported to cleave type I collagen and attenuate collagen deposition by HSC, inhibiting liver fibrosis (Radbill et al., 2011). As to TIMPs, they could inhibit or activate fibrosis via MMPs. For example, TIMP-3 inhibits MMPs to induce inflammation (Gill et al., 2010) in lung injury, and TIMP-1 has been found to play a dual role in liver fibrosis (Wang H. et al., 2011).

Other extracellular factors include a wide range of proteins and peptides. They mainly guide the differentiation of myofibroblasts, and are closely related to each other and growth factors. For example, the hedgehog (Hh) signaling pathway mediates EMT during the fetal development, and responds to injury through the repression of epithelial marker epithelial-cadherin by *Snail* and *Twist*. Overactivation of Hh signaling pathway contributes to biliary fibrosis and related liver fibrosis (Omenetti et al., 2008). In fibrotic kidney, the upregulated Wnt signaling has been reported to result in abundant of β-catenin. The signaling regulates genes such as *Twist, LEF1* to induce EMT, thus to aggravate disease (He et al., 2009).

### Intracellular factors mediating the progression of fibrosis

Intracellular factors, mainly multiple kinases, propagate the signaling received by cells through phosphorylation and other pathways. A common consequence of the signaling is that, transcription activators or inhibitors translocate into nucleus to regulate fibrosis-related gene expression and cell responses. In certain inflammatory pathways, intracellular factors also modulate the expression of extracellular factors, such as growth factors and cytokines, and secrete them out of cells to amplify inflammatory responses. Besides, epigenetic factors are emerging as a new way to affect fibrosis-related gene expression.

Intracellular factors include a number of kinases. In inflammation phase, many upstream factors, such as TGF-β, TNF-α, and epidermal growth factors initiate mitogen-activated protein kinase (MAPK) pathway. An element of MAPK pathway, mitogen-activated protein kinase-activated protein kinase-2 mediates myofibroblasts differentiation and regulates the gene expression of several matrix proteins such as *col1a2, col3a1*, and *lox* (Vittal et al., 2013). Another important intracellular signaling, mechanistic target of rapamycin (mTOR) pathway is activated by Wnt and TNF-α. Then, mTOR pathway activates ribosomal protein S6 kinase β-1 and modulates protein p21 or p27, which regulates the cell cycle of many cells including fibroblasts. In addition, the inhibition of mTOR pathway reduces collagen deposition and cardiac fibrosis (Chen et al., 2012). Besides serving as amplifiers of signal transduction, some intracellular enzymes such as cathepsin K, which belongs to lysosomal cysteine proteases, have been proved to be able to degrade ECM in lysosome after phagocytosis (Fukumori et al., 2003; Buhling et al., 2004).

Nuclear receptors are receptors located in cytoplasm and nucleus that could receive signals from intracellular ligands and bind to DNA to regulate gene expression. For example, peroxisome proliferator activated receptor γ(PPAR-γ), may directly regulate type I collagen gene (Yang et al., 2006) and block TGF-β signaling (Ghosh et al., 2009). Another nuclear receptor, farnesoid-X receptor (FXR), exhibits anti-fibrotic effect via the reduction of proliferating cholangiocytes and subsequent reduction of TGF-β (Liu et al., 2003). The activation of FXR also decreases a series of pro-fibrotic factors including TIMP-1, collagens, α-SMA, and MMP-2 (Zhang et al., 2009).

Nowadays, epigenetics including microRNAs, DNA methylation and lncRNAs, are found involved in machinery of pro-fibrotic process mainly through regulating fibrosis-related gene expression. Some microRNAs are found to negatively regulate translation of ECM components. Among them, miR-21 induces extracellular-signal regulated kinase/MAPK activity via the inhibition of *Spry1* to protect cardiac fibroblasts survival (Thum et al., 2008). It has been reported that the levels of DNA methylation at specific CpG sites of pro-fibrotic genes (*PPAR*α*, PPAR*δ*, TGF*β*1, Collagen1A1*, and *PDGF*α) differ among different fibrosis stages in NAFLD (Zeybel et al., 2015).

There are factors that affect fibrosis process both extra- and intracellularly, including reactive oxygen species (ROS), galectin-3 and lysyl oxidase homolog 2 (LOXL2). ROS can be generated through tissue injuries, cell damages and NADPH oxidase activities. Extracellular ROS targets latency-associated peptides and then activates TGF-β signaling while intracellular oxidative stress induces p53-dependent apoptosis in lung fibrosis via the caspases-9/3 activation in mitochondria (Cheresh et al., 2013). As to galectin-3, extracellular galectin-3 induces T cell apoptosis and plays a dual function inside and outside cells (Li et al., 2014). Another factor LOXL2 is generally considered as extracellular enzyme that promotes collagen production and crosslink with collagen fibers in response to mechanical stress (Yang et al., 2016). While on the other hand, intracellular LOXL2 has been reported to induce EMT in carcinoma progression (Peinado et al., 2005).

Many studies proved that different fibroproliferative diseases share common underlying mechanisms (Wenzke et al., 2012). The existence of common mechanisms facilitates the complete interpretation of fibrosis pathogenesis and enhances our understanding of fibrosis-related diseases. On this basis, it is necessary to reconsider targets involved in these mechanisms and evaluate their potential roles in fibrosis treatment across tissues and organs.

## Drugs and targets in fibrosis

Motivated by huge clinical burdens, continuous intense researches on drug targeting fibrosis have been conducted, many of which have led to clinical trials. Due to the strong associations between inflammation and fibrosis, more efforts have been devoted to anti-inflammation drugs in the past few years (Dinwiddie, 2005). Nowadays, new targets and drugs for fibrosis are constantly emerging with the progress in understanding fibrosis pathology. Here we summarize them in Tables 1–**4**, including single (Tables 1–**3**) and multi component (**Table 4**) drugs with their verified and potential targets in fibrosis.

**Table 1:** Single-component drugs targeting extracellular factors.

**Target**				**Drug**			**Clinical trial**^b^		**Reference**
**Group**	**Target or mechanism type**	**Target or mechanism**	**Organs^a^**	**Drug Name**	**Mechanism**	**Class**	**Disease**	**Phase**	**Reference/Trial identifier^c^**
Growth factors	Extracellular TGF-β signaling	TGF-β^d^	Liver, Kidney, Lung, Heart, Pancreas, Skin, Gut	SHP-627 (FT011)	Inhibitor	Small molecule	Cardiac fibrosis	Preclinical	Zhang et al., 2012
				Hydronidone (F351)	Inhibitor	Small molecule	Liver fibrosis	2(unknown)	NCT02499562
				PXS-25	Inhibitor	Small molecule	IPF^d^	Preclinical	Maldonado et al., 2009; Wong et al., 2011
				Disitertide (P-144)	Inhibitor	Small molecule	Skin fibrosis	2(completed)	NCT00574613
				Fresolimumab (GC-1008)	Inhibitor	Monoclonal antibody	IPF; SSc^d^	1(completed); 1(completed)	NCT00125385; NCT01284322
				LY2382770	Inhibitor	Monoclonal antibody	Diabetic kidney disease	2(terminated)	NCT01113801
		Integrin αvβ6		STX-100	Inhibitor	Monoclonal antibody	IPF	2(completed)	NCT01371305
				CWHM-12	Inhibitor	Small molecule	Liver fibrosis; Lung fibrosis	Preclinical	Henderson et al., 2013
		ALK5^d^		SB-431542	Antagonist	Small molecule	Pulmonary fibrosis	Preclinical	Koh et al., 2015
		BMP-7^d^		THR-184	Agonist	Small molecule	Renal fibrosis	2(completed)	NCT01830920
	CTGF^d^	CTGF		PF-06473871	Inhibitor	Small molecule	Hypertrophic scar	2(completed)	NCT01730339
				RXI-109	Inhibitor	Small molecule	Hypertrophic scar	2(completed)	NCT02030275
				FG-3019	Inhibitor	Monoclonal antibody	IPF	2(active, not recruiting)	NCT01890265
	PDGF^d^/VEGF^d^	PDGFR^d^		Imatinib	Antagonist	Small molecule	Nephrogenic systemic fibrosis; SSc; IPF	Approved| 2(completed); 2(completed); 3(completed)	NCT00677092; NCT00613171; NCT00131274
				BOT-191	Antagonist	Small molecule	Liver fibrosis	Preclinical	van Dijk et al., 2015
				Nilotinib (AMN-107)	Antagonist	Small molecule	SSc	Approved| 2(completed)	NCT01166139
				Dasatinib	Antagonist	Small molecule	Scleroderma pulmonary fibrosis	Approved| 2(completed)	NCT00764309
		VEGFR^d^/PDGFR		Nintedanib (BIBF-1120)	Antagonist	Small molecule	Scleroderma; IPF	Approved| 3(recruiting); 3(completed)	NCT02597933; NCT01335464
				Sorafenib (BAY 43-9006)	Antagonist	Small molecule	Extensive keloids	Approved| 2(terminated)	NCT01425216
	TNF^d^	TNF		Thalidomide	Inhibitor	Small molecule	IPF	Approved| 2(completed)	NCT00162760
				Pomalidomide	Inhibitor	Small molecule	IPF	Approved| 2(withdrawn)	NCT01135199
				Etanercept	Inhibitor	Recombinant protein	IPF	Approved| 2(completed)	NCT00063869
				Belimumab	Inhibitor	Monoclonal antibody	SSc	Approved| 2(completed)	NCT01670565
	HGF^d^	HGF	Liver, Kidney, Lung, Heart, Skin	Refanalin (BB-3)	Stimulant	Small molecule	Liver fibrosis; IPF	Preclinical	Fallowfield, 2011
Cytokines	Interleukin	IL-13^d^	Liver, Kidney, Lung, Heart, Pancreas, Skin, Gut	Dectrekumab (QAX-576)	Inhibitor	Monoclonal antibody	IPF; IPF secondary to SSc	2(terminated); 2(terminated)	NCT01266135; NCT00581997
				Tralokinumab	Inhibitor	Monoclonal antibody	IPF	2(terminated)	NCT01629667
		IL-1R1	Liver, Kidney, Lung, Heart, Skin, Gut	Anakinra	Antagonist	Recombinant protein	Cystic fibrosis	Approved| Preclinical	Iannitti et al., 2016
		IL-1βR		Rilonacept	Antagonist	Recombinant protein	SSc	Approved| 2(active, not recruiting)	NCT01538719
		IL-13/IL-4		SAR156597	Inhibitor	Monoclonal antibody	SSc; IPF	2(recruiting); 2(completed)	NCT02921971; NCT01529853
	CC chemokine	CCL2^d^	Liver, Kidney, Lung, Heart, Pancreas, Skin, Gut	Carlumab (CNTO-888)	Inhibitor	Monoclonal antibody	IPF	2(completed)	NCT00786201
				Bindarit	Inhibitor	Small molecule	Myocardial fibrosis; Renal fibrosis	Preclinical	Lin et al., 2009; Zhu et al., 2009
		CCR5^d^	Liver, Kidney, Lung	Maraviroc	Antagonist	Small molecule	Liver fibrosis	Approved| Preclinical	Gonzalez et al., 2014
		CCR2	Liver, Kidney, Lung, Heart, Pancreas, Skin, Gut	RS-504393	Antagonist	Small molecule	Renal fibrosis	Preclinical	Kitagawa et al., 2004
	Interferon	IFN-γR^d^		Actimmune	Stimulant	Interferon	IPF; Liver fibrosis; Cystic fibrosis	Approved| 3(completed); 2(completed); 2(completed)	NCT00047658; NCT00043303; NCT00043316
		IFN-α	Liver, Kidney, Lung	Interferon alpha oral lozenge	Stimulant	Interferon	Pulmonary fibrosis	2(completed)	NCT01442779
MMP^d^/TIMP^d^	MMP/TIMP	MMP-2/MMP-9/TIMP-1	Liver, Kidney, Lung, Heart, Pancreas, Skin, Gut	Batimastat(BB-49)	Inhibitor	Small molecule	IPF	Preclinical	Corbel et al., 2001
		MMP/TIMP		Marimastat	Inhibitor	Small molecule	Liver fibrosis	Approved| Preclinical	de Meijer et al., 2010
Other proteins and peptides	Endothelin	ET-1 receptor^d^	Liver, Kidney, Lung, Heart, Skin, Gut	Macitentan	Antagonist	Small molecule	IPF	Approved| 2(completed)	NCT00903331
				Bosentan	Antagonist	Small molecule	IPF; SSc;	Approved| 3(completed); 3(completed); 4(completed)	NCT00070590; NCT00319696; NCT01395732
				Ambrisentan	Antagonist	Small molecule	IPF; SSc	Approved| 3(terminated); 4(unknown)	NCT00879229; NCT01051960
				Sparsentan (RE-021)	Antagonist	Small molecule	Focal segmental glomerulosclerosis	2(active, not recruiting)	NCT01613118
				Atrasentan	Antagonist	Small molecule	Renal fibrosis	Preclinical	Samad et al., 2015
	Angiotensin II	AT1 receptor^d^	Liver, Kidney, Lung, Heart, Pancreas, Skin, Gut	Losartan	Antagonist	Small molecule	Liver fibrosis; Cystic fibrosis	Approved| 4(completed); 2(not yet recruiting)	NCT00298714; NCT03206788
	GPCR^d^	LPAR^d^	Liver, Kidney, Lung, Skin	BMS-986020	Antagonist	Small molecule	SSc; IPF	2(withdrawn); 2(completed)	NCT02588625; NCT01766817
				SAR-100842	Antagonist	Small molecule	SSc	2(completed)	NCT01651143
		PAR1^d^	Liver, Kidney, Lung, Heart, Pancreas, Skin	PAR1 antagonism	Antagonist	Small molecule	Liver fibrosis	Preclinical	Fiorucci et al., 2004
		CB1 receptor^d^	Liver	Curcumin^*^	Antagonist	Small molecule	Liver fibrosis; Renal fibrosis; IPF	Preclinical	Smith et al., 2010; Zhang et al., 2013; Sun et al., 2017
				Silymarin^*^	Antagonist	Small molecule	Liver fibrosis	Preclinical	Tsai et al., 2008; Zhang et al., 2013
		CB2 receptor^d^		β-caryophyllene^*^	Agonist	Small molecule	Liver fibrosis	Preclinical	Calleja et al., 2013; Mahmoud et al., 2014
		Prostacyclin receptor	Liver, Kidney, Lung, Heart, Pancreas	Beraprost	Agonist	Small molecule	Renal fibrosis; Cardiac fibrosis	Preclinical	Chen et al., 2014
				Iloprost	Agonist	Small molecule	SSc	Approved| 2(completed)	NCT00109681
				Treprostinil	Agonist	Small molecule	IPF; SSc	Approved| 2(terminated); 2(completed)	NCT00703339; NCT00775463
		VIP receptor	Lung	Aviptadil	Agonist	Peptide hormone	Cystic fibrosis	Preclinical	Mathioudakis et al., 2013
	Leukocyte elastase	Leukocyte elastase		Sivelestat	Inhibitor	Small molecule	IPF	Preclinical	Takemasa et al., 2012
	TAFI^d^	TAFI	Liver, Kidney, Lung	UK-396082	Inhibitor	Small molecule	Renal fibrosis	Preclinical	Atkinson et al., 2015
	Relaxin	Relaxin receptor	Liver, Kidney, Lung, Heart, Skin	Serelaxin	Stimulant	Peptide hormone	Cardiac fibrosis; Renal fibrosis	Preclinical	Samuel et al., 2014; Huuskes et al., 2015
	SAP^d^	SAP (mimic)		PRM-151	Stimulant	Recombinant protein	IPF	2(active, not recruiting)	NCT02550873
	Integrin α	Integrin α5	Liver	Dioscin^*^	Inhibitor	Small molecule	Liver fibrosis	Preclinical	Liu et al., 2015; Zhang et al., 2015a,b; Gu et al., 2016; Xu et al., 2017; Yin et al., 2017
	TGM^d^	TGM2	Kidney, Lung	NTU281	Inhibitor	Small molecule	Renal fibrosis	Preclinical	Johnson et al., 2007

* *Drug belongs to monomer extracted from natural products*.

a *Organs that had study report of corresponding targets in fibrosis treatment*.

b *Clinical trial resource are from http://Clinicaltrials.gov*.

c Trial Identifier is the clinical trail identifier of corresponding drug

d *TGF-β, transforming growth factor-β; IPF, idiopathic pulmonary fibrosis; SSc, systematic sclerosis; ALK5, TGF-β receptor 1; BMP-7, bone morphogenetic protein 7; CTGF, connective tissue growth factor; PDGF, platelet-derived growth factor; PDGFR, platelet-derived growth factor receptor; VEGF, vascular endothelial growth factor; VEGFR, vascular endothelial growth factor receptor; TNF, tumor necrosis factor; HGF, hepatocyte growth factor; IL-13, interleukin-13; CCL2, chemokine (C-C motif) ligand 2; CCR5, C-C chemokine receptor type 5; IFN-γR, interferon-γ receptor; MMP, matrix metalloproteinase; TIMP, tissue inhibitor of metalloproteinase; ET-1 receptor, endothelin-1 receptor; AT1 receptor, angiotensin II receptor type 1; GPCR, G protein-coupled receptor; LPAR, lysophosphatidic acid receptor; PAR1, protease-activated receptor 1; CB1 receptor, cannabinoid receptor type 1; TAFI, thrombin activatable fibrinolysis inhibitor; SAP, serum amyloid P; VIP, vasoactive intestinal peptide; TGM2, transglutaminase*.

### Single-component drugs targeting extracellular factors mediating fibrosis

Nowadays, most approved and investigational drugs are single-component drugs, which only contain one organic component and have distinct targets. As we described previously, fibrosis progression results from a combination of the abnormalities of extracellular and intracellular factors. Drugs targeting extracellular factors are prevalent, about 60% of known targets are receptors located on cell membrane mainly because the extracellular targets are accessible and serve as upstream signals (Overington et al., 2006), and so are the targets of fibrosis-related drugs. The binding of receptors and ligands triggers the downstream signaling, thus the blockade of receptors or ligands is considered to be an effective choice to alleviate fibrosis (Table 1).

The majority of anti-fibrosis drugs targeting extracellular factors are inhibitors of ligands such as growth factors, cytokines and MMPs. Most inhibitors could directly bind to the active sites of targets. The majority of approved inhibitor drugs in Table 1 target TNF, which are widely used in fibrosis-related diseases and could suppress the action of TNF through multiple mechanisms. These drugs include small molecules Thalidomide and Pomalidomide (Weingartner et al., 2012), recombinant protein Etanercept and monoclonal antibodies Belimumab. Thalidomide and Etanercept have completed the phase II trial in IPF (Raghu et al., 2008; Horton et al., 2012) while Belimumab has completed the phase II trial in SSc. Besides, some inhibitor drugs targeting growth factors are still under investigation. For example, Disitertide, a synthetic peptide derived from TGF-β type III receptor, inhibits the binding of TGF-β and its receptor and exhibit anti-fibrotic function (Ezquerro et al., 2003). Drugs that inhibit interleukins are always monoclonal antibodies. Tralokinumab, a human IgG4 monoclonal antibody, shows pro-apoptotic effects via IL-induced apoptotic factors in IPF (Murray et al., 2014). Many natural products act as inhibitor drugs like Dioscin, which is a monomer extracted from *Dioscoreae Rhizoma* and could ameliorate liver fibrosis (Liu et al., 2015; Zhang et al., 2015a,b; Gu et al., 2016; Xu et al., 2017; Yin et al., 2017). In addition, some preclinical drugs inhibit MMPs, like Marimastat, which simultaneously down-regulates MMPs gene expression and MMPs activities. However, Marimastat reduces inflammation and liver injuries while increases fibrosis in mice model. This may result from the indiscriminative inhibition of MMPs, some of which function to degrade ECM (de Meijer et al., 2010).

Compared with inhibitor drugs, antagonists achieve the same inhibition effect by targeting cell membrane receptors to dampen downstream signaling. These small molecules bind to receptors without effectively activating them. The receptors of PDGF, vascular endothelial growth factor, endothelin (ET), and angiotensin all have approved antagonist drugs. An antagonist for tyrosine kinase receptors of PDGF, Imatinib, has showed protective effect by reducing differentiation of resting fibroblasts in SSc mice model (Akhmetshina et al., 2009). However, it did not show efficacy in phase II clinical trial in IPF (Daniels et al., 2010) and the high-dose of Imatinib may lead to severe adverse events (Khanna et al., 2011). Macitentan, a dual antagonist of ET_A_ and ET_B_ receptor, is beneficial for lung fibrosis. Similarly, another drug Losartan targeting angiotensin II receptor has been evaluated in IPF patients (Couluris et al., 2012). Some preclinical antagonist drugs including Maraviroc (Gonzalez et al., 2014), Atrasentan (Ritter et al., 2014), and PAR1 antagonists (Fiorucci et al., 2004) were under investigation for fibrosis treatment.

On the contrary, many drugs exert their therapeutic effects by activating their targets. Many anti-fibrotic receptors can be targets of these exogenous agonists that augment the downstream biological responses to suppress fibrosis. An approved agonist drug in this category is Iloprost, which can reverse right ventricle fibrosis by re-establishing collagen balance (Gomez-Arroyo et al., 2015). Another agonist of vasoactive intestinal peptide, Treprostinil, reduces inflammation and collagen deposition (Manitsopoulos et al., 2015). Other anti-fibrotic agonists for cell membrane receptors include Aviptadil, INT-767 (Baghdasaryan et al., 2011) and Beraprost (Kaneshige et al., 2007).

Moreover, a few drugs are synthetic proteins that bind receptors to serve as stimulants and perform the same functions as native proteins. An approved drug, synthetic interferon-γ, Actimmune, has completed phase II or phase III study in multiple fibrosis including IPF (Skaria et al., 2015), liver fibrosis (Muir et al., 2006), and cystic fibrosis (Moss et al., 2005). Another stimulant Refanalin, a HGF mimetic, is a potential drug for liver fibrosis (Fallowfield, 2011; Pellicoro et al., 2014).

### Single-component drugs targeting intracellular factors mediating fibrosis

Compared with extracellular factors, intracellular targets are less popular owing to their inaccessibility. Drugs targeting intracellular factors are less varied because most of them are small molecules. Small molecules could readily translocate into cytoplasm while large molecules such as monoclonal antibodies face more challenges to cross the plasma membrane (Imai and Takaoka, 2006). Nevertheless, more and more studies concerned intracellular factors as targets in recent years, and numerous candidate targets are identified in cytoplasm, nucleus, and mitochondrion. Many approaches, including increasing membrane permeation, combination with supercharged proteins and activating transport through receptors, were implemented to deliver drugs across cell membrane (Mitragotri et al., 2014).

Fibrosis drugs targeting intracellular factors are summarized and classified into four categories: enzymes, nuclear receptors, other proteins, and epigenetics (Table 2). Many drugs acting through intracellular factors are also inhibitors. These drugs inhibit a wide range of kinases located in cytoplasm, and consequently suppress the translocation of transcription factors that drive the expression of pro-fibrotic genes. Rapamycin and Sirolimus are approved drugs that inhibit mTOR. Rapamycin prevents the activation of macrophages and myofibroblasts and the subsequent release of TGF-β in chronic kidney disease (CKD) (Chen et al., 2012). Sirolimus shows anti-inflammatory and anti-fibrotic effects in IPF (Tulek et al., 2011). Besides kinases, there are many other intracellular proteins that serve as potential targets for fibrosis management. Pirfenidone, one of the IPF drugs, has completed phase III trial in IPF patients with alleviated disease progression and acceptable side effects (King et al., 2014). The potential mechanism of Pirfenidone is inhibiting the nuclear accumulation of intracellular proteins SMAD2/3 to regulate TGF-β signaling (Choi et al., 2012). Other approved inhibitor drugs include Ruxolitinib for bone marrow fibrosis (Wilkins et al., 2013), Paquinimod for SSc (Stenstrom et al., 2016), and Pentoxifylline (Okunieff et al., 2004) combined with vitamin E (Jacobson et al., 2013).

**Table 2:** Single-component drugs targeting intracellular factors.

**Target**				**Drug**			**Clinical trial**^b^		**Reference**
**Group**	**Target or mechanism type**	**Target or mechanism**	**Organs^a^**	**Drug Name**	**Mechanism**	**Class**	**Disease**	**Phase**	**Reference/Trial identifier^c^**
Enzymes	mTOR^d^	mTORC1/2^d^	Liver, Kidney, Lung, Heart, Skin, Gut	Rapamycin (Sirolimus)^*^	Inhibitor	Small molecule	Renal interstitial fibrosis	Approved| 3(completed)	NCT01079143
				Palomid-529 (RES-529)	Inhibitor	Small molecule	Macular degeneration	1(completed)	NCT01033721
	JAK-STAT^d^	JAK1/JAK2^d^		Ruxolitinib	Inhibitor	Small molecule	Myelofibrosis	Approved| 3(completed)	NCT00952289
				Baricitinib	Inhibitor	Small molecule	Renal interstitial fibrosis	Preclinical	Breyer and Susztak, 2016
	PI3K-Akt^d^	Akt	Liver, Kidney, Lung, Heart, Skin	Omipalisib (GSK2126458)	Inhibitor	Small molecule	IPF^d^	1(completed)	NCT01725139
		FAK1^d^	Liver, Kidney, Lung, Heart, Pancreas, Skin	PF-562271	Inhibitor	Small molecule	Pulmonary fibrosis; Cardiac fibrosis; Liver fibrosis	Preclinical	Lagares et al., 2012; Fan et al., 2015; Zhao et al., 2017
	MAPK^d^	JNK^d^		Tanzisertib (CC-930)	Inhibitor	Small molecule	IPF	2(terminated)	NCT01203943
		MAPK	Liver, Kidney, Lung, Heart, Pancreas, Skin, Gut	MMI-0100	Inhibitor	Small molecule	IPF; Cardiac fibrosis	Preclinical	Xu et al., 2014
	NF-κB^d^	IKK^d^		IMD-1041	Inhibitor	Small molecule	Cardiac fibrosis	Preclinical	Tanaka et al., 2012
				Bardoxolone methyl (CDDO-Me)	Inhibitor	Small molecule	Pulmonary hypertension	2(recruiting)	NCT02036970
		NF-κB		Antisense NF-κB	Inhibitor	Antisense oligonucleotide	Intestinal fibrosis	Preclinical	Lawrance et al., 2003
				Baicalein^*^	Inhibitor	Small molecule	Renal fibrosis; IPF	Preclinical	Gao et al., 2013; Wang et al., 2015
				Sulfasalazine	Inhibitor	Small molecule	Liver fibrosis; Pancreatic fibrosis	Approved| Preclinical	Chavez et al., 2012; Wang et al., 2016
	cAMP-PKA^d^	ROCK^d^		Y-27632	Inhibitor	Small molecule	Renal fibrosis; Liver fibrosis	Preclinical	Tada et al., 2001
	Non-kinase enzyme	26S protease	Liver, Kidney, Lung, Heart	Bortezomib	Inhibitor	Small molecule	SSc pulmonary fibrosis	Approved| 2(recruiting)	NCT02370693
		Caspase	Liver, Kidney, Lung, Heart, Skin	Emricasan	Inhibitor	Small molecule	Liver fibrosis	Preclinical	Barreyro et al., 2015
				VX-166	Inhibitor	Small molecule	Liver fibrosis	Preclinical	Witek et al., 2009
				Z-VAD-fmk	Inhibitor	Small molecule	Pulmonary fibrosis	Preclinical	Kuwano et al., 2001
		PDE^d^	Kidney, Heart	CTP-499	Inhibitor	Small molecule	Diabetic nephropathy	1(completed)	NCT01328821
		Cathepsin B	Liver, Lung, Heart, Pancreas	VBY-376	Inhibitor	Small molecule	Liver fibrosis	Preclinical	Alkhouri et al., 2011
				CA-074Me	Inhibitor	Small molecule	Pancreatic fibrosis; Cardiac fibrosis; Pulmonary fibrosis	Preclinical	Lerch and Halangk, 2006; Liu et al., 2013; Zhang et al., 2015
		S100A9	Liver, Lung, Heart, Skin	Paquinimod	Inhibitor	Small molecule	SSc^d^	2(completed)	NCT01487551
		Procollagen-proline dioxygenase	Liver, Lung	HOE-077	Inhibitor	Small molecule	Liver fibrosis	Preclinical	Matsumura et al., 1997
Nuclear receptors	PPAR^d^	PPAR-γ	Liver, Kidney, Lung, Heart, Pancreas, Skin, Gut	Rosiglitazone	Agonist	Small molecule	Liver fibrosis;	Approved| 2(completed)	NCT00492700
				Elafibranor (GFT-505)	Agonist	Small molecule	Liver fibrosis	3(recruiting)	NCT02704403
				Saroglitazar	Agonist	Small molecule	Liver fibrosis	2(recruiting)	NCT03061721
				Pioglitazone	Agonist	Small molecule	Cystic fibrosis; Liver fibrosis	Approved| 1(completed); 1(completed)	NCT00719381; NCT01454336
				Docosahexaenoic acid^*^	Agonist	Small molecule	Liver fibrosis; Pulmonary fibrosis	Preclinical	Depner et al., 2013; Zhao et al., 2014
	FXR^d^	FXR	Liver, Kidney, Lung, Gut	INT-767	Agonist	Small molecule	Liver fibrosis	Preclinical	Baghdasaryan et al., 2011
				PX-102	Agonist	Small molecule	Liver fibrosis	Preclinical	Ali et al., 2015
				Obeticholic acid^*^ (INT-747)	Agonist	Small molecule	Liver fibrosis	Approved| 3(recruiting)	NCT02548351
				Turofexorate isopropyl (WAY-362450)	Agonist	Small molecule	Liver fibrosis	Preclinical	Zhang et al., 2009
				GW4064	Agonist	Small molecule	Liver fibrosis	Preclinical	Liu et al., 2003
	GR^d^	GR	Liver, Lung, Heart, Skin	Triamcinolone	Agonist	Small molecule	Keloid scarring	Approved| 1(terminated)	NCT01978301
	ER^d^	ERβ	Liver	Genistein^*^	Agonist	Small molecule	Pulmonary fibrosis; Liver fibrosis	Preclinical	Salas et al., 2008; Nadadur et al., 2012
Other proteins	Intracellular TGF-β^d^ signaling	SMAD2/3	Liver, Kidney, Lung, Heart, Pancreas, Skin, Gut	Pirfenidone	Inhibitor	Small molecule	IPF; SSc	Approved| 3(completed); 2(completed)	NCT00287729; NCT01933334
		SMAD3/4		Pentoxifylline	Inhibitor	Small molecule	Skin fibrosis	Approved| 2(completed)	NCT00001437
		SMAD3		SIS-3	Inhibitor	Small molecule	Renal fibrosis	Preclinical	Meng et al., 2015
				Glycyrrhizin^*^	Inhibitor	Small molecule	Liver fibrosis	3(terminated)	NCT00686881
Epigenetics	miRNA	miR-21		Anti-miR-21	Inhibitor	Oligonucleotide	IPF; Renal fibrosis	Preclinical	Liu et al., 2010; Chau et al., 2012
	methylation	Transmethylation	Liver, Kidney, Lung, Heart, Skin, Gut	Ademetionine (SAM)	Inhibitor	Small molecule	Liver fibrosis	Approved| Unknown	NCT02231333
		BMPER (gene)	Lung	DNA methylation	Inhibitor	Methylation	IPF	Preclinical	Huan et al., 2015

* *Drug belongs to monomer extracted from natural products*.

a *Organs that had study report of corresponding targets in fibrosis treatment*.

b *Clinical trial resource are from http://Clinicaltrials.gov*.

c *Trial Identifier is the clinical trail identifier of corresponding drug*.

d *mTOR, mechanistic target of rapamycin; mTORC1, mechanistic target of rapamycin complex 1; JAK-STAT, janus kinase/signal transducers and activators of transcription; PI3K-Akt, phosphoinositide 3-kinase/protein kinase B; IPF, idiopathic pulmonary fibrosis; FAK1, focal adhesion kinase 1; MAPK, mitogen-activated protein kinase; JNK, c-Jun N-terminal kinases; NF-κB, nuclear factor kappa-light-chain-enhancer of activated B cells; IKK, I-kappa B kinase; cAMP-PKA, cyclic AMP- protein kinase A signaling; ROCK, rho-associated protein kinase; PDE, phosphodiesterase; SSc, systematic sclerosis; PPAR, peroxisome proliferator-activated receptor; FXR, farnesoid X receptor; GR, glucocorticoid receptor; ER, estrogen receptor; TGF-β, transforming growth factor-β*.

In addition, there are also some nuclear receptors located in cytoplasm and nucleus, which could be activated by small molecule agonists, such as PPAR. Rosiglitazone, a PPAR-γ agonist, has anti-fibrotic effect as a consequence of activating MMP-1 and elevating HGF expression in patients with systemic sclerosis-related interstitial lung disease (Bogatkevich et al., 2012). Other approved PPAR targeting drugs like Elafibranor and Pioglitazone, have entered phase II and phase I studies, respectively. Obeticholic acid is an approved drug entering phase III study that decreases inflammation and fibrosis in NASH patients via activating FXR signaling (Verbeke et al., 2016).

Epigenetics are very different types of fibrosis therapies. The most studied epigenetics-based therapy for fibrosis is microRNA. MicroRNA could be neutralized by specific anti-miRNA oligonucleotides delivered into cells. Among them, anti-miR-21 has been reported to inhibit miR-21 activity and ameliorate fibrosis progression through PPAR signaling in CKD (Chau et al., 2012). Another strategy is the intervention of DNA methylation for proteins such as BMP endothelial cell precursor-derived regulator (BMPER), which acts as the regulator of fibroblasts activation. Altering methylation on *BMPER* gene has been reported to decrease BMPER level and thus to inhibit fibroblasts activity (Huan et al., 2015). Besides, some drugs targeting both extracellular and intracellular factors are also incorporated (Table 3). A majority of them are antioxidants, including an approved drug N-acetylcysteine (Zhang et al., 2014).

**Table 3:** Single-component drugs targeting both extra- and intracellular factors.

**Target**			**Drug**			**Clinical trial**^b^		**Reference**
**Target or mechanism type**	**Target or mechanism**	**Organs^a^**	**Drug Name**	**Mechanism**	**Class**	**Disease**	**Phase**	**Reference/Trial identifier^c^**
LOX^d^	LOXL2^d^	Liver, Kidney, Lung, Heart, Skin, Gut	β-aminopropionitrile (BAPN)	Inhibitor	Small molecule	Cardiac fibrosis	Preclinical	Martinez-Martinez et al., 2016
			Simtuzumab (GS-6624)	Inhibitor	Monoclonal antibody	Liver fibrosis; IPF^d^	2(completed); 2(terminated)	NCT01452308; NCT01769196
ROS^d^	NOX1^d^/NOX4	Liver, Kidney, Lung, Heart, Pancreas, Skin	GM-CT-01	Inhibitor	Polymer	Liver fibrosis	Preclinical	Traber and Zomer, 2013
			GR-MD-02	Inhibitor	Polymer	Liver fibrosis	2(completed)	NCT02421094
			GCS-100	Inhibitor	Polymer	Renal fibrosis	2(completed)	NCT01843790
	ROS		GKT137831	Inhibitor	Small molecule	Liver fibrosis	Preclinical	Aoyama et al., 2012
			N-acetylcysteine^*^	Inhibitor	Small molecule	IPF	Approved| Preclinical	Demedts et al., 2005; Zhang et al., 2014
			Mitoquinone	Inhibitor	Small molecule	Liver fibrosis	Preclinical	Vilaseca et al., 2017
			Salvianolic acid B^*^	Inhibitor	Small molecule	Liver fibrosis; Renal fibrosis; IPF	Preclinical	Liu et al., 2002, 2016; Pan et al., 2011
			Resveratrol^*^	Inhibitor	Small molecule	Liver fibrosis	3(completed)	NCT02030977
	Vitamin (mimic)	Liver, Kidney, Lung, Heart, Pancreas, Skin, Gut	Pyridoxamine	Inhibitor	Small molecule	Renal fibrosis	2(completed)	NCT00320060
			α-tocopherol	Inhibitor	Small molecule	IPF	Approved| Preclinical	Deger et al., 2007
	Collagen (mimic)	Liver, Kidney, Lung, Skin	IW001	Inhibitor	Collagen	IPF	1(completed)	NCT01199887

* *Drug belongs to monomer extracted from natural products*.

a *Organs that had study report of corresponding targets in fibrosis treatment*.

b *Clinical trial resource are from http://Clinicaltrials.gov*.

c *Trial Identifier is the clinical trail identifier of corresponding drug*.

d *LOX, lysyl oxidase; LOXL2, lysyl oxidase homolog 2; IPF, idiopathic pulmonary fibrosis; ROS, reactive oxygen species; NOX1, NADPH oxidase 1*.

### Multi-component drugs used for fibrosis

Differed from single-component drugs that target a single protein or other simple targets, multi-component drugs contain more than one active ingredient. Traditional Chinese medicines (TCM) therapies, usually appeared as herbal formula, have been studied for thousands of years as multi-component drugs (Wang et al., 2012). Nowadays, single compound acting on multiple targets and multiple compounds acting on multiple targets are popular strategies in drug development (Hopkins, 2008). Fibrosis includes numerous complicated pathological pathways. Multi-component drugs, aiming at different targets, have the advantage in modulating these pathways simultaneously and producing synergistic effects. Moreover, multi-component drugs are expected to provide great resources for discovering new effective drug molecules. Many studies have revealed the pharmacology of multi-component drugs in the fibrosis treatment (Feng et al., 2009; Yang et al., 2009; Li and Kan, 2017) (Table 4). For example, Fuzhenghuayu capsule (FZHY), a well-known multi-component drug for treating liver fibrosis, inhibits liver fibrosis and improves liver function in patients via inhibition of nuclear factor kappa-B kinase subunit β/nuclear factor κF and TGF-β signaling (Liu et al., 2005). Another emerging multi-component drug, Qishenyiqi (QSYQ), is under phase II clinical trial for ischemic heart failure. QSYQ attenuates cardiac fibrosis via IL-6/STAT3 and TNF-α/nuclear factor kappa-light-chain-enhancer of activated B cells (NFκB) signalings and anti-apoptosis activities (Wang et al., 2017). Multi-component drugs act on different physiological reactions associated with fibrosis, such as inflammation and angiogenesis, leading to a systematic improvement of disease. Qushi Huayu Decoction (QHD) is a multitargeting drug that alleviates fibrosis by reducing ROS via the induction of glutathione and modulating lipid metabolism and gut barrier function (Feng et al., 2017).

**Table 4:** Multi-component drugs.

**Drug**		**Clinical trial**^a^		**Reference**
**Drug Name**	**Mechanism**	**Disease**	**Phase**	**Reference/Trial identifier^b^**
Fuzhenghuayu capsule (FZHY)	TGF-β^c^/MMP-2^c^	Liver fibrosis	2(completed); 4(recruiting)	NCT00854087; NCT02241616
Qishenyiqi (QSYQ)	TNF^c^/TGF-β/β-Catenin	Ischemic heart failure	2(recruiting)	NCT02875639
Qushi Huayu Decoction (QHD)	ROS^c^	Liver fibrosis	Preclinical	Feng et al., 2017
Herbal compound 861 (Cpd 861)	TGF-β/MMP-1/TIMP-1^c^	Liver fibrosis	Preclinical	Hou et al., 2016
Xiao-Chai-Hu Tang (XCHT)	IL-6^c^/TNF-α/Bax protein	Liver cancer	2(completed)	NCT00040898; Zhou et al., 2012
Dahuangzhechong pill (DHZCP)	α-SMA^c^/TNF-α/IL-13/p38 MAPK^c^/ERK^c^	Liver fibrosis	Preclinical	Cai et al., 2010
Han-dan-gan-le	ROS/collagen	Liver fibrosis	Preclinical	Li et al., 1998
*Qianggan-Rongxian* Decoction	−	Liver fibrosis	Preclinical	Li et al., 2008
Yi-gan-kang granule	type I collagen/TIMP-1	Liver fibrosis	Preclinical	Yao et al., 2005
Ginkgo biloba extract	TGF-β	Liver fibrosis	Preclinical	Ding et al., 2005
Rosa laevigata Michx (RLTS)	ROS/CYP2El^c^/TGF-β/SMAD/FAK^c^-PI3K^c^-Akt^c^-p70S6K^c^/MAPK	Liver fibrosis	Preclinical	Dong et al., 2015
Liuweiwuling (LWWL) tablets	TGF-β/SMAD/ NF-κB^c^	Liver fibrosis	Preclinical	Liu et al., 2017
Xuefuzhuyu (XFZY) decoction	HIF-Y^c^/DDAH^c^/ADMA^c^/VEGF^c^	Liver fibrosis	Preclinical	Zhou et al., 2014
Diwu Yanggan (DWYG)	TGF-β/BMP-7^c^	Liver fibrosis	Preclinical	Shen et al., 2014
Ocimum gratissimum extracts (OGEs)	ROS/α-SMA	Liver fibrosis	Preclinical	Chiu et al., 2014
Yin-Chiao-San (YCS)	ROS/TNF-α	IPF^c^	Preclinical	Yen et al., 2007
Renshen pingfei decoction	TGF-β/SMAD3	IPF	Preclinical	Chen et al., 2016
Hu-qi-yin	TGF-β	IPF	Preclinical	Zhou et al., 2007
Decoction for Strengthening Qi and Replenishing Lung (DSQRL)	–	IPF	Preclinical	Zhang et al., 2008
Modified Kushen Gancao Formula (mKG)	TGF-β/IL-6/IL-17A	IPF	Preclinical	Gao et al., 2016
Sho-seiryu-to (TJ-19)	ROS	IPF	Preclinical	Yang et al., 2010
Hochu-ekki-to (TJ-41)	IL-5/IL-4/IFN-γ	IPF	Preclinical	Tajima et al., 2007
Shenlong Decoction	MMsP/TIMPs	IPF	Preclinical	Lu et al., 2010
Yupingfeng	HMGB1^c^/TGF-β	IPF	Preclinical	Cui et al., 2015
Danggui–Buxue–Tang (DBTG)	TNF-α/TGF-β	IPF	Preclinical	Lv et al., 2012

a *Clinical trial resource are from http://Clinicaltrials.gov*.

b *Trial Identifier is the clinical trail identifier of corresponding drug*.

c *TGF-β, transforming growth factor-β; MMP-2, matrix metalloproteinase 2; TNF, tumor necrosis factor; ROS, reactive oxygen species; TIMP, tissue inhibitor of metalloproteinase; IL-6, interleukin-6; α-SMA, α-smooth muscle actin; MAPK, mitogen-activated protein kinase; ERK, extracellular signal-regulated kinase; PDGF-BB, platelet-derived growth factor-BB; IPF, idiopathic pulmonary fibrosis; HMGB1, high mobility group box 1; CYP2E1, cytochrome P450 2E1; FAK, focal adhesion kinase; PI3K, phosphatidylinositol-3-kinase; Akt, amino kinase terminal; p70S6K, 70-kDa ribosomal S6 Kinase; NF-κB, nuclear factor kappa-light-chain-enhancer of activated B cells; HIF-α, hypoxia inducible factors; DDAH, dimethylarginine dimethylaminohydrolase; ADMA, asymmetric dimethylarginine; VEGF, vascular endothelial grow factor; BMP-7, bone morphogenetic protein 7*.

With multiple targets being discovered, it becomes apparent that more common targets will be shared across many fibrotic diseases with common mechanisms, though some proteins will not express in special conditions. Meanwhile, there will be more chance for drug repositioning, which indicate common drugs will be shared across different targets andfibroproliferative diseases.

## Perspective

Fibrosis is a common pathological process in many diseases, causing a great clinical burden in recent years. The development of state-of-the-art technologies facilitate discovery of fibrosis therapies. Multi-omics analysis provides a more convenient and systematic way for researching on disease mechanisms (Fernandes and Husi, 2017). The seamless combination of traditional transcriptomics approaches with emerging technologies, including proteomics (Ordureau et al., 2014), metabolomics (Shah et al., 2012), and metagenomics (Jiao et al., 2017), will offer unprecedented opportunities to precisely elucidating and dissecting fibrosis mechanisms. Nowadays, the application of cryo-electron microscopy (cryo-EM) in macromolecular structure determination make it easier to identify drug targets (Zheng et al., 2015). Compared with traditional X-ray crystallography, cryo-EM has advantage in determining the structure of more complex and flexible receptors (Huang et al., 2016; Zhang et al., 2017). Finally, with the advent of the era of big data, artificial intelligence technology, especially deep learning, provides more accurate algorithms for drug repositioning (LeCun et al., 2015; Alaimo et al., 2016). The continuous development and application of the above technologies and methods will make it possible to identify and discover more common mechanisms, targets and drugs in fibrosis.

## Author contributions

RZ and LZ conceived and designed the project. Each author has contributed significantly to the submitted work. XL drafted the manuscript. LZ, BW, MY, and RZ revised the manuscript. All authors read and approved the final manuscript.

### Conflict of interest statement

The authors declare that the research was conducted in the absence of any commercial or financial relationships that could be construed as a potential conflict of interest. The reviewer XT and handling Editor declared their shared affiliation.

## Abbreviations

ECM: extracellular matrix
IPF: idiopathic pulmonary fibrosis
NAFLD: non-alcoholic fatty liver disease
TGF-β: transforming growth factor-β
IL-6: interleukin-6
SSc: systemic sclerosis
PDGF: platelet derived growth factor
EMT: epithelial-mesenchymal transition
HSC: hepatic stellate cell
MMPs: matrix metalloproteinases
α-SMA: α-smooth muscle actin
HGF: hepatocyte growth factor
TNF: tumor necrosis factor
STAT3: signal transducer and activator of transcription 3
TIMPs: tissue inhibitor of metalloproteinases
Hh signaling: hedgehog signaling
mTOR: mechanistic target of rapamycin
MAPK: mitogen-activated protein kinase
PPAR-γ: peroxisome proliferator activated receptor γ
FXR: farnesoid-X receptor
LOXL2: lysyl oxidase homolog 2
ROS: reactive oxygen species
CKD: chronic kidney disease
ET: endothelin
BMPER: BMP endothelial cell precursor-derived regulator
TCM: traditional Chinese medicines
FZHY: Fuzhenghuayu capsule
NFκB: nuclear factor kappa-light-chain-enhancer of activated B cells
QSYQ: Qishenyiqi
QHD: Qushi Huayu Decoction.
cryo-EM: cryo-electron microscopy.
